# Acoustic Emission of Deformation Twinning in Magnesium

**DOI:** 10.3390/ma9080662

**Published:** 2016-08-06

**Authors:** Chengyang Mo, Brian Wisner, Mike Cabal, Kavan Hazeli, K. T. Ramesh, Haitham El Kadiri, Talal Al-Samman, Konstantin D. Molodov, Dmitri A. Molodov, Antonios Kontsos

**Affiliations:** 1 Theoretical & Applied Mechanics Group, Department of Mechanical Engineering, Drexel University, Philadelphia, PA 19104, USA; cm963@drexel.edu (C.M.); bjw63@drexel.edu (B.W.); mcabal1988@gmail.com (M.C.); 2 Hopkins Extreme Material Institute, John Hopkins University, Baltimore, MD 21218, USA; hazeli@jhu.edu (K.H.); ramesh@jhu.edu (K.T.R.); 3 Department of Mechanical Engineering, Mississippi State University, Starkville, MS 39762, USA; elkadiri@me.msstate.edu; 4 Institute of Physical Metallurgy and Metal Physics, RWTH Aachen University, Aachen 52062, Germany; Alsamman@imm.rwth-aachen.de (T.A.-S.); Molodov@imm.rwth-aachen.de (K.D.M.); kmolodov@imm.rwth-aachen.de (D.A.M.)

**Keywords:** Acoustic Emission, twinning, Magnesium

## Abstract

The Acoustic Emission of deformation twinning in Magnesium is investigated in this article. Single crystal testing with combined full field deformation measurements, as well as polycrystalline testing inside the scanning electron microscope with simultaneous monitoring of texture evolution and twin nucleation were compared to testing at the laboratory scale with respect to recordings of Acoustic Emission activity. Single crystal testing revealed the formation of layered twin boundaries in areas of strain localization which was accompanied by distinct changes in the acoustic data. Testing inside the microscope directly showed twin nucleation, proliferation and growth as well as associated crystallographic reorientations. A post processing approach of the Acoustic Emission activity revealed the existence of a class of signals that appears in a strain range in which twinning is profuse, as validated by the in situ and ex situ microscopy observations. Features extracted from such activity were cross-correlated both with the available mechanical and microscopy data, as well as with the Acoustic Emission activity recorded at the laboratory scale for similarly prepared specimens. The overall approach demonstrates that the method of Acoustic Emission could provide real time volumetric information related to the activation of deformation twinning in Magnesium alloys, in spite of the complexity of the propagation phenomena, the possible activation of several deformation modes and the challenges posed by the sensing approach itself when applied in this type of materials evaluation approach.

## 1. Introduction

Magnesium (Mg) has a hexagonal close-packed (hcp) structure (c/a=1.624) and a limited number of easy slip systems compared to fcc metals [1]. Basal 〈a〉 glide, in addition to 〈a〉 prism and pyramidal slip systems, which, however, require higher driving forces and/or elevated temperatures for activation, are known to exist. To accommodate c-axis strains, pyramidal 〈c+a〉 slip systems and deformation twinning can occur [2,3,4]. Twins can be both of extension and contraction type, and unlike slip, they generally cause anisotropic changes of the initial crystallographic grain orientation (texture), which depends on the applied forming procedure, the process followed to prepare samples/components and the imposed direction of loading [5]. When a growing twin meets a grain boundary it produces a well-defined tilt that appears as the “needle” structure within the grains. Input load reversibility has been associated with detwinning (i.e., twin annihilation) activity [6,7], which causes additional changes in texture and is related to the observed pseudoelasticity of Mg alloys [8]. Twinning has been further related to redistributions of internal stress/strain that cause: (i) higher order twinning (e.g., daughter twins in grains that had previously formed twins, i.e., parents) [9,10], activation of multiple twin variants [2,11] and double-twinning [4,12]; (ii) activation of “harder” slip systems [8] (e.g., pyramidal 〈c+a〉); and (iii) interactions with slip systems that could lead to grain-scale microcracks, which accumulate with loading and ultimately cause fatigue failure [4]. The crystallographic deformation mechanisms in addition to single and polycrystal dislocation-defect interactions in Mg alloys determine their distinct macroscopic mechanical behavior, including asymmetric tension/compression yielding and anisotropic strain hardening [13], which complicate forming procedures and create the need for detailed microstructure-sensitive characterization.

Various methods have been applied to study deformation twinning in Magnesium. Using texture analysis [14,15,16,17,18], {10$\bar{1}$2} twins were observed in single crystal compression [19] while other forms of twins were observed under higher temperature [20]. In addition, heterogeneous extension twins were observed when conducting Channel-Die compression of single crystals [21]. The activation of extension twins and non-basal slips were additionally observed with Transmission Electron Microscope (TEM) by performing nanoindentation and microcompression [22,23]. Electron backscattered diffraction (EBSD) analyses revealed favorable orientations for twinning in Mg polycrystals [24]. Furthermore, twin boundary mobility has been also studied using in situ Scanning Electron microscopy (SEM) observations to calculate twin boundary velocity [25].

Acoustic Emission (AE) is a non-destructive investigation method used, among other applications, in material deformation and damage investigations [13,26,27,28,29,30]. AE activity occurs in all materials under applied loading and can be formally defined as transient elastic waves generated by rapid release of energy caused by events at various length scales, such as crack initiation and propagation, fiber fracture, delaminations, stress corrosion, fatigue and creep. In the case of metals, AE activity has been recorded from dislocation motion/glide [29,30,31,32,33,34], twinning [13,29,35,36], yielding [37], hardening [13] and phase transformations [38]. At the macroscale, the travelling stress waves related to AE are detected even at the small-scale damage level, i.e., long before final macroscopic failure, by specially designed piezoelectric transducers, as well as with a variety of other sensors (e.g., fiber Bragg) and are converted to voltage versus time waveforms. The inverse problem of damage identification based on AE has been greatly enhanced over the past years with the application of mathematical methods, such as neural networks and clustering algorithms that are used to parameterize, identify correlations and statistically group information in pattern recognition algorithms [26,39,40]. Therefore, AE is a diagnostic tool with the potential for the characterization of the mechanical behavior of advanced materials with hierarchical microstructures and multiple phases, in various loading conditions including fatigue, since it can: (i) monitor reversible/irreversible microstructural changes across time and length scales; and (ii) detect dynamic effects, such as dislocation motion and crack initiation. 

Various attempts have been made to understand twinning in Mg using AE. Studies have been conducted for AZ [13,41,42], ZK [43] and AM [36] alloys, showing the correspondence between peak of AE count rate and the yield point on stress strain curves. In addition, the effect of grain size and solute content on the AE response was revealed by several studies [44,45,46]. An asymmetry in the AE activity was reported by multiple investigations due to dependence on the loading direction and crystal orientation [36,47]. Furthermore, recent approaches of coupling microstructure and AE have been introduced to support the relationship between AE response and twin nucleation [48,49]. Furthermore, the AE activity during Mg cyclic tests showed the asymmetry of AE response in different loading conditions [34,50,51]. Moreover, machine learning techniques, such as clustering and neural network analyses, have been used to discriminate AE signals from different sources, such as dislocation motions and twin nucleation [52,53]. A similar machine learning approach was used for TRIP/TWIP steels to separate different deformation mechanisms such as stacking faults and martensitic phase transformation [40,54,55]. It should be added here that, apart from work with polycrystalline Mg, only one study is known to the authors that has performed AE monitoring on Mg single crystals [47].

In this article, an approach is proposed to record the AE activity generated by twin nucleation in Mg. To achieve this goal, a comprehensive study of Mg twinning is conducted across length scales and materials, including both single crystal and polycrystals. Digital Image correlation (DIC), scanning electron microscopy (SEM), and electron backscattered diffraction (EBSD) were additionally used to verify the relation of recorded AE signals with activation of deformation twinning. Machine learning techniques were also used to identify characteristics of AE signals generated from twinning. The presented results relate for the first time to the authors best knowledge the AE activity of twin nucleation in single crystals with in situ observed twinning in polycrystals and associated twin-related AE information using laboratory scale specimens.

## 2. Experimental Procedure

### 2.1. Mechanical and Nondestructive Evaluation Setup

**Figure 1:**
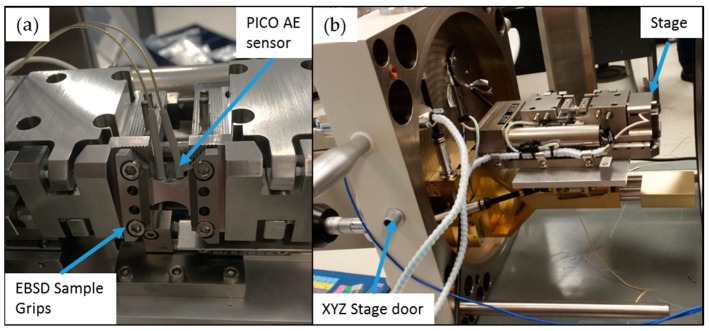
Experimental mechanics setup including a screw-driven stage used for tensile testing of Magnesium polycrystals inside the Scanning Electron Microscope: (**a**) The loading stage with sensors and sample attached on the EBSD grip which is pre-tilted 70°; (**b**) The XYZ stage door with stage on the door.

Three experimental mechanics setups targeting twin activation across different scales coupled with simultaneous recordings of AE activity were used in this investigation. Specifically, Figure 1 shows the first setup consisting of a mechanical stage used for tensile tests of Mg AZ31 polycrystals inside the SEM. The stage is a screw driven Gatan MTEST with a 2000 N load cell which was placed inside an FEI XL30 SEM. A custom built sample grip pre-tilted to 70° was further used for in situ EBSD measurements. The samples were loaded on the stage with a load rate of 0.1 mm/min. EBSD data were collected with EDAX/TSL system at accelerated voltage of 30 kV, spot size 6, aperture size of 100 μm, and working distance of 15 mm with step size of 2 μm. EBSD analysis was performed using EDAX/TSL OIM analysis software. EBSD mapping and SEM images were taken while the samples were held at constant strain.

A MTS universal testing system at room temperature was used for the other two setups including compression tests on Mg single crystals at a constant strain rate of 5 × 10^−4^·s^−1^ and tension of Mg AZ31 specimens with a constant strain rate of 4.5 × 10^−4^·s^−1^. Speckle patterns were applied on both the single crystal and polycrystal specimens for strain mapping through the DIC method. 3D DIC results were obtained from a dual camera setup using the commercially available ARAMIS software, as previously shown by the authors in the case of full field deformation measurements of Mg alloys [50,56,57,58].

The AE setup used for all three mechanical testing procedures described above consisted of a AE data acquisition board, a pre-amplifier, and a piezoelectric sensor as described in previous work by the authors [59], while it is worth mentioning that it was the same across all experimental setups, which allows for direct comparisons across scales, specimens, and types of tests. Specifically, AE signals were recorded using PICO piezoelectric sensors, the PCI-2 Acquisition board, and 2/4/6 pre-amplifiers all commercially available by Mistras Group Inc. The AE and DIC systems were linked to load cell voltage readouts which allowed their time synchronization, as shown previously by the authors [60]. 

### 2.2. Sample Preparation

**Figure 2:**
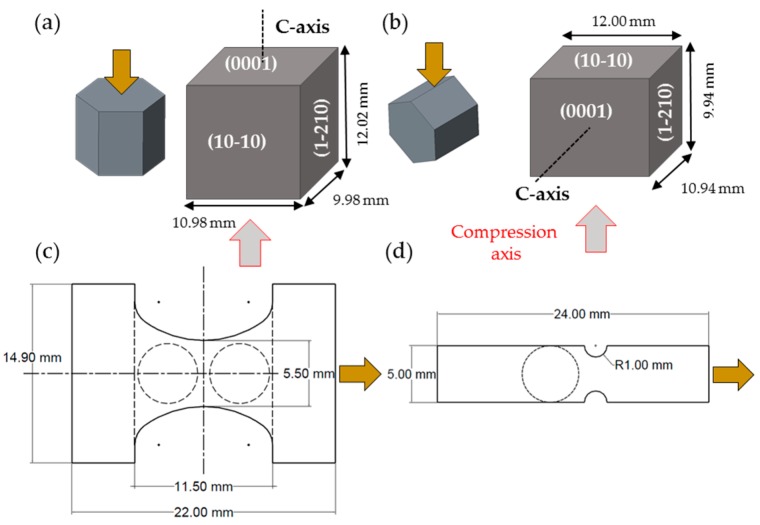
Specimen geometry of: Magnesium single crystal for compression along (**a**) the *c*-axis and (**b**) along the *a*-axis; tensile specimens of Magnesium AZ31 used for (**c**) testing inside the SEM and (**d**) using a MTS frame.

Three different types of Mg specimens were tested in this investigation as shown in Figure 2. Magnesium single crystals (Figure 2a,b) were grown in a steel mold under argon atmosphere as described by Molodov [24]. Testing cubes were cut from such single crystals in two orientations for compression testing using a MTS frame. Compression along the c-axis or a-axis was coupled with simultaneous DIC measurements on the corresponding face of the test cubes indicated by the arrows in Figure 1 and AE monitoring using one sensor.

**Figure 3:**
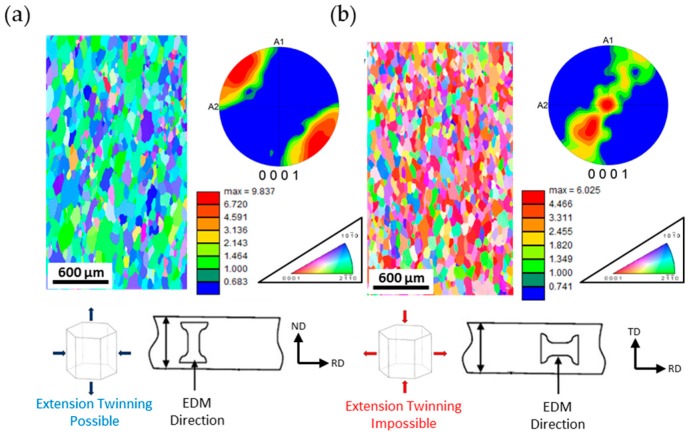
Microstructure of tensile specimens: EBSD map and corresponding pole figures of: (**a**) ND orientation; (**b**) RD orientation.

The tensile samples used for testing inside the SEM chamber (Figure 2c) and with a MTS frame (Figure 2d) were produced from a commercial AZ31 alloy rolled plate, as shown in Figure 3. Cubes were cut out from the 25.4 mm thick plate and were compressed in the Normal Direction (ND) up to 5% compressive strain to increase the dislocation density and stimulate grain growth, as previously described by the authors [50,56]. The cubes were then heat treated at 500 °C for 50 h for grain growth. Larger grains were pursued to lower the activation stress of twinning and to make twinning more observable using the SEM. After heat treatment, samples with a thickness of 2 mm were machined from the cube using Electrical Discharged Machining (EDM). The samples for in situ testing were designed to fit in the stage of Figure 1 with the added capability to attach up to two AE sensors. The samples were ground and then polished with 6 μm, 3 μm, and 1 μm diamond suspension. A mirror finish was achieved with 0.05 μm alumina suspension. The samples were then etched by immersing in a solution of 5% nitric acid, 15% acetic acid, 20% distilled water, and 60% ethanol for 3 s. The initial microstructures of micro tensile samples are shown in Figure 3. EBSD and texture analysis showed that the RD samples had a predominantly basal texture and therefore were not favorable to deform by extension twinning. ND samples had a prismatic texture and therefore were possible to deform by extension twinning. Both types of samples had an average grain size of 100 μm.

## 3. Results and Discussion

### 3.1. Single Crystal Compression

**Figure 4:**
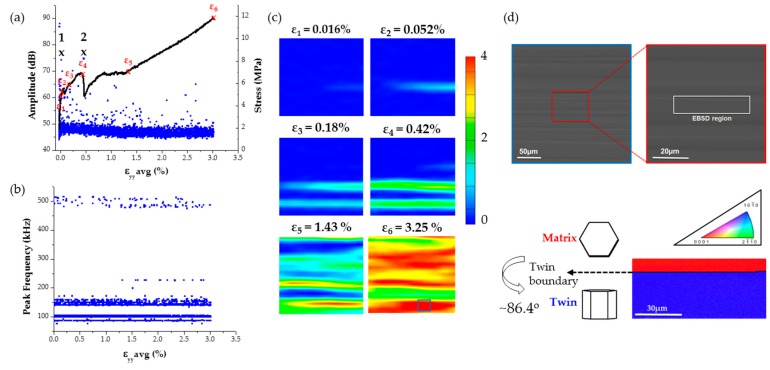
(**a**) Stress-strain curve for a-axis compression specimen coupled with the distribution of the AE waveform amplitudes; (**b**) corresponding distribution of AE signal peak frequency values; (**c**) full field strain maps along the loading direction and (**d**) region definition for EBSD measurements and schematic that explains the formation of a twin boundary.

The stress-strain curve combined with the amplitude distribution of the AE signals for an *a*-axis single crystal compression test is shown in Figure 4a. The peak frequency distribution of the AE waveforms whose amplitude distribution is shown in Figure 4a, is plotted Figure 4b. The corresponding full field strains along the loading direction (named *y*-axis for reference) are shown in Figure 4c. It should be noted that the numerical values of strain used in the stress-strain curve of Figure 4a correspond to average of the strain maps shown in Figure 4c. Overall the stress-strain curve appears to have a sigmoidal shape, which is characteristic of twinning activity, marked by a strain plateau that initiates at strain ε_3_ and extends up to strain ε_5_ [50,57]. This type of load drops agree with previous study [61] that reports load drops during compression of single crystals. This study further suggested that such load drops are possibly caused by dislocation-solute and dislocation-dislocation interactions which can be associated with Lüders band propagation or serrations on the stress-strain curve. In fact, the authors using a continuum plasticity model recently showed that such load drops could indeed be caused by shear band formation [62].

Most of the AE signals in this test were found to have amplitude values between 40 and 50 dB. However, some higher amplitude (>65 dB) AE signals were observed at strain value ε_2_ = 0.052% at which point the stress-strain curve appears to show a small load drop followed by a slope change. Interestingly, at this strain value a horizontal strain localization appears as shown in Figure 4c. This strain localization intensifies and a new localization band parallel to the one observed at ε_2_ appears at strain ε_3_, followed by a more significant now load drop at strain value ε_4_,which is accompanied by a second jump of the AE amplitude distribution, as shown in Figure 4a. Past strain ε_4_ the stress increases again non-linearly with respect to the average strain along the loading direction, while at strain ε_5_ (=1.43%) the slope of this curve increases. Past strain ε_5_ the full field strain maps appear to have multiple localization zones until failure at strain ε_6_.

Strain localization bands during monotonic and cyclic loading of Mg polycrystals were first reported by the authors [50,56,57,58] and others [63,64], while their appearance was spatially correlated with the inhomogeneous activation of twinning [57]. Based on such prior information and since to the authors best knowledge this is the first time that such results are reported for single crystals of Mg, a region was selected based on the electron microscopy images shown in Figure 4d to perform EBSD measurements. The EBSD scan revealed the presence of extension twinning as confirmed by the measured misorientation angle of 86.4°. As a result, there is evidence to suggest that the strain localizations seen in the DIC strain maps correspond to twinning activity which in this case appears to create a horizontal twin boundary as explained schematically in Figure 4d. 

**Figure 5:**
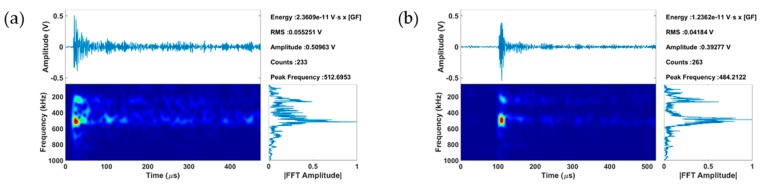
Two identified AE signals related to initiation of twinning in *a*-axis compression of Mg single crystals: (**a**) Signal 1 identified in Figure 4a; (**b**) Signal 2 identified in Figure 4a.

Two extracted features of the recorded AE signals that are identified in Figure 4 include the distinct high amplitude values at given time/strain instances during this monotonic compression test, as well as the appearance of relatively high (~500 kHz) peak frequency values, which both agree with reports found in previous investigations [48,65]. The coupling of such AE data with full field strains allows the direct correlation of such features with the initiation of a clearly defined strain localization band, as seen in Figure 4c. Moreover, load drops are observed exactly at loading increments were the high amplitude AE waveforms were recorded. This observation agrees with a previous investigation [61] that reports load drops after twin nucleation. Hence, AE signals, such as the ones shown in Figure 5, which were recorded in instances when bulk load drops, are very possibly directly related to initiation of twinning in Mg single crystals. These AE signals consist of burst type waveforms with distinct peak frequency at ~500 kHz confirmed by wavelet transform, which also corresponds to the sharp increase of amplitude that occurs at the time window defined by these waveforms.

**Figure 6:**
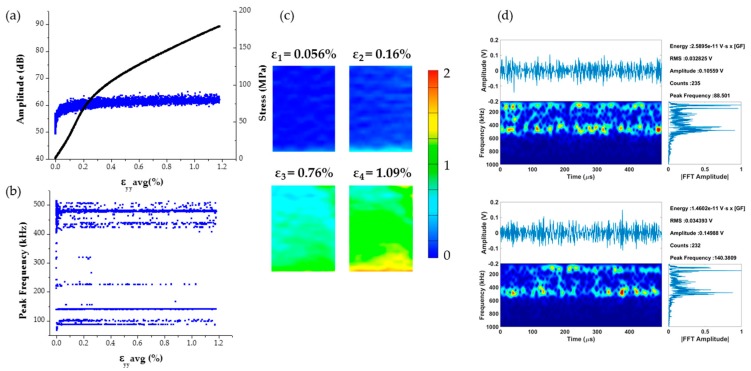
(**a**) Stress-strain curve for c-axis compression specimen coupled with the distribution of the AE waveform amplitudes; (**b**) corresponding distribution of AE signal peak frequency values; (**c**) full field strain maps along the loading direction; (**d**) randomly selected AE signals.

To compare the results shown in Figure 4 with *c*-axis compression data, Figure 6 shows the stress–strain curve coupled with AE amplitude distribution and corresponding full field strain data. In this case, the stress-strain curve is a smoothly nonlinear typical of a ductile metal, while the AE response shows no outliers with all related signals having amplitude between 50 and 65 dB. In addition, no strain localization in the DIC strain maps is observed in this case until near the end of the experiment when high strains were observed near the edges of the specimens coinciding with the locations of the compression platens used to apply the load. Two randomly-selected AE waveforms from this experiment are shown in Figure 6d, which shows that most AE signals from *c*-axis compression are of continuous type and hence different compared to the burst type signals recorded during a-axis compression and shown in Figure 5.

### 3.2. AZ31 Tensile Tests Inside the SEM 

**Figure 7:**
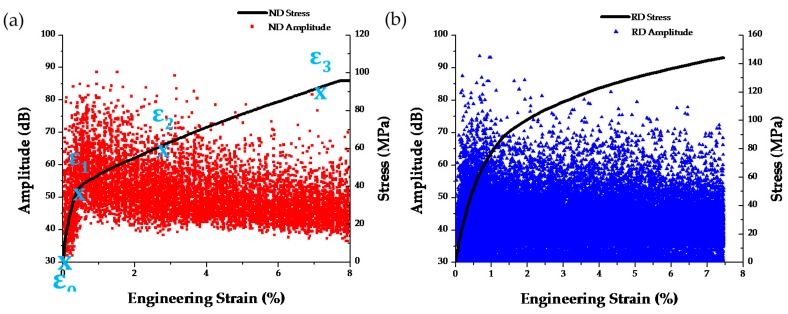
Stress strain curve and corresponding AE amplitude distribution of tensile tests of: (**a**) ND and (**b**) RD specimens.

The corresponding stress-strain curve coupled with the amplitude distribution of AE signals in the case of a tensile test of AZ31 specimens cut along both the ND and RD directions are shown in Figure 7. Similar to the single crystal tests, anisotropic mechanical behavior is observed for the two types of samples. The ND sample has a yield stress of ~50 MPa, while the RD sample has a yield stress of ~60 MPa. Due to the geometry of the specimens, slippage was observed at the grips when high load were reached and therefore the specimens did not fail. High amplitude AE signals were only observed near yield points for both samples which have similar maximum amplitudes. It should be further noted that in order to monitor the evolution of texture and the initiation of twinning, DIC measurements were not performed in these experiments and, therefore, the strain values in Figure 7 correspond to engineering strain computed given the displacement readings by the stage.

**Figure 8:**
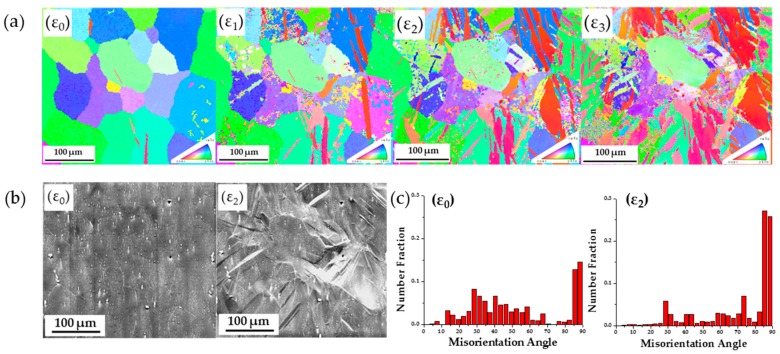
(**a**) Texture evolution and twin activity monitoring of the ND specimen at corresponding strain values marked in the stress-strain curve of Figure 7; (**b**) SEM images at corresponding strain values ε_0_ and ε_2_; (**c**) misorientation angle distribution between 0 and 90 degrees at corresponding strain values ε_0_ and ε_2_.

To compare the AE response in Figure 7 in these two tests, direct microstructure monitoring using EBSD measurements and SEM images is shown in Figure 8. The ND specimen shows severe twinning activity while the RD samples does not. Specifically, in situ obtained EBSD maps at the indicated strain values on the stress-strain curve of the ND sample are shown in Figure 8a. It can be clearly seen that the original microstructure shows a relatively twin free texture with some remnant twins due to the thermomechanical processing. At strain value ε_1_ twins appear in several grains. Further twin nucleation and growth are observed in the previously twinned grains at ε_2_. At ε_3_, some grains are fully reoriented to basal texture. SEM images of the same area before and after the test are shown in Figure 8b. Surface extrusion and intrusion after twin nucleation are observed, as described previously by the authors [56]. The distribution of misorientation angle at ε_0_ and ε_2_ is shown in Figure 8c, from which extension twinning can be confirmed by the fact that several occurrences of misorientation angle between 85 and 90 degrees is found. The misorientation angle is calculated between identified grains with grain tolerance angle of 5 degrees.

**Figure 9:**
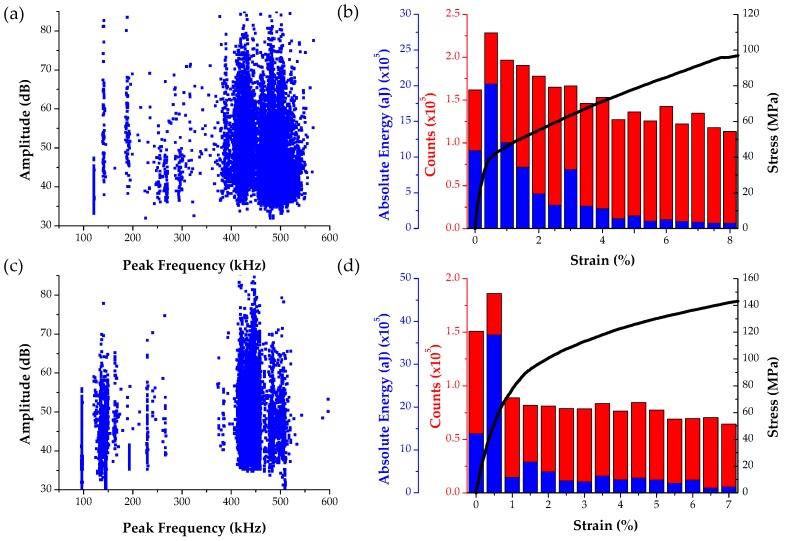
Analysis of the AE activity of the specimens tested inside the SEM: (**a**,**c**) amplitude vs. peak frequency plot and; (**b**,**d**) distribution of AE counts and absolute energy for ND (**top**) and RD (**bottom**) specimens.

Further analysis of the AE results shown in Figure 7 was performed to identify specific information related to the twinning activity. Specifically, a comparison of the peak frequency values shown in Figure 9 indicates that the ND specimen has more AE signals with dominant frequency in the 450–500 kHz range and less signals in the 100–200 kHz. This is expected, as Li [65] also showed that a twinning AE signal will have a peak frequency around 490 kHz. Note that the AE signals in the single crystal tests previously reported in Figure 5 also have such a peak frequency in this range, which provides additional evidence that this frequency-based AE feature is useful to flag the initiation of twinning activity.

Distributions of AE counts and absolute energy of AE signals as a function of engineering strain are shown in Figure 9b,d. The AE counts and absolute energy distributions peak near the yield point. It has been shown that AE responses are most intensive near yield point in Magnesium due to twin nucleation and massive dislocation multiplication [41,42,66]. In the ND direction, twinning should be the major source of AE signal during the early stages of deformation, as also supported by the direct microstructure results shown in Figure 8. In contrast, in RD samples dislocation motion should be the main source of AE signal as extension twins are not possible for this crystal orientation. In addition, it can be further observed that the AE counts and absolute energy decrease after yielding. However, such a drop is much more rapid in the RD compared to the ND specimen. This observation could be explained by the fact that, in the ND specimen, twin nucleation should be prominent near the macroscopically defined yield point, which explains the peak in both counts and absolute energy for burst type emissions such as the ones reported in Figure 5. The slow drop in counts and absolute energy past yielding could be attributed, based also on the in situ obtained results in Figure 8, to the fact that twins are still nucleating, growing and interacting after the yield point. Consequently, the counts and absolute energy distributions present a rather slow decrease until ~3%, which previous work by the authors has shown that it corresponds to the end of the plateau and the onset of hardening in the stress strain curve of specimens prone to twinning [57].

**Figure 10:**
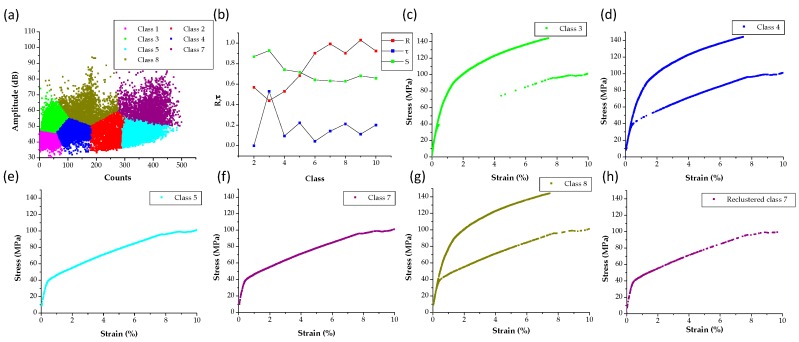
(**a**) K-Mean clustering results based on the combined AE datasets of both ND and RD tests that have a Peak Frequency between 350 and 550 kHz; (**b**) computed *R*, τ and S values against number of classes; (**c**–**g**) class 3–5 and 7–8 AE hits plotted as a projection on the ND and RD stress strain curves; (**h**) reclustered class 7 AE hits.

To further classify differences in the AE data for the specimen tested inside the SEM, a clustering approach was followed based on the use of the K-means method with the following features of amplitude, AE counts, absolute energy, and peak frequency according to prior work of the authors [26,28] and based on the AE signals shown in Figure 7 and Figure 9. Clustering analysis is a pattern recognition technique which groups data according to their similarity. Eight classes were identified for signals with peak frequency between 350 and 550 kHz which is the frequency range in which both single crystal and SEM tests have showed that twinning AE signals exist, as shown in Figure 10a. The optimum number of classes was selected using three performance indices, namely the *R*, τ, and *S* values, where *R* is the Davies-Bouldin index, τ is the minimum cluster center distance divided by maximum intercluster distance as defined by Tou [67], while *S* is the Sihouette index. In general, low values for *R* and high value of τ and *S* are desired for the clustering to be well-defined. The computed *R*, τ, and *S* values as a function number of classes are plotted in Figure 10b. Even though a three class clustering appears to have the highest τ and *S* and lowest *R* value, it does not produce a class of AE signals that were only existent in the ND direction which was desirable. On the other hand, the eight class clustering shown in Figure 10a, has a local minimum in *R* and a local maximum for τ as shown in Figure 10b. Therefore, it could be argued that clustering with eight classes not only produces an acceptable classification based on the three criteria used in this investigation, but it also leads to two classes, class 5 and class 7, which exist only in the AE dataset of the ND specimen, as shown by plotting the AE data in their projection in the corresponding stress–strain curves shown in Figure 10c–g. Note that such projection was possible, due to the synchronization between AE, DIC and load data which allows the so called “hit-driven” projection of AE data using two external to the AE system parameters, in this case stress and strain values. A comparison between class 5 and class 7 data shows that AE signals in class 5 generally have lower amplitudes compared to class 7 data. Although the authors are well aware that amplitude is not a reliable classifier in such analyses, the high amplitude of the signals in class 7 combined with their larger number of counts (as shown in Figure 10a), the results in Figure 9 (which confirms large number of counts for twinning AE activity) and both the single crystal (Figure 4) and SEM test data (Figure 7) previously reported, lead to the possibility of these signals to be representative of AE information from twinning.

**Figure 11:**
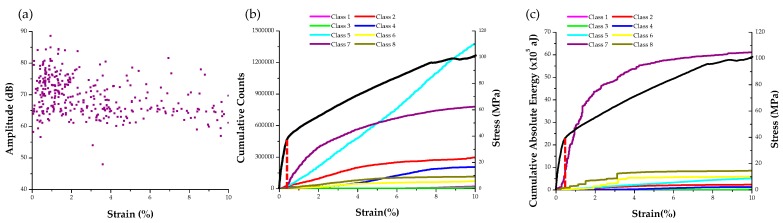
(**a**) Amplitude vs. strain of the reclustered class 7; (**b**,**c**) cumulative AE counts and absolute energy of all classes as a function of strain and compared with the stress–strain curve of the ND specimen.

Further analysis of all AE waveforms within class 7 revealed both continuous and burst type emissions. To further classify the burst type signals, which are more possible to originate from twinning activity, as shown in Figure 5, a new feature extraction with adaptive threshold was performed on all AE signals within this class. Subsequently the data were clustered again using the features of duration and risetime, motivated by the fact that burst type signals by definition have short duration and risetime. The reclustered AE hits are projected on the ND load curve shown in Figure 10h. In addition, the amplitude vs. strain of the reclustered class 7 signals is shown in Figure 11a. Interestingly, this projection of the clustered data shows that most of the AE signals in this class occur during early stages of the test (<3%). Moreover, Figure 11b,c show that AE signals in class 7 have much larger number of counts and the highest absolute energy compared to all other classes in the strain range that in Figure 8 was directly associated with profuse twinning. In addition, the cumulative absolute energy of this reclustered dataset appears to increase drastically near the yield point. This is consistent with the results in Figure 9b, which suggested that signals with the most energy can be found near the yield point. The AE information of the signals within the reclustered class 7 is, therefore, both statistically and physically meaningful to be correlated with twinning activity in Mg. To further evaluate such information, the AE activity of tension tests using a standard MTS frame for the specimens shown in Figure 2c was further analyzed, as shown in the following figures.

### 3.3. AZ 31 Tensile Tests Using the MTS 

**Figure 12:**
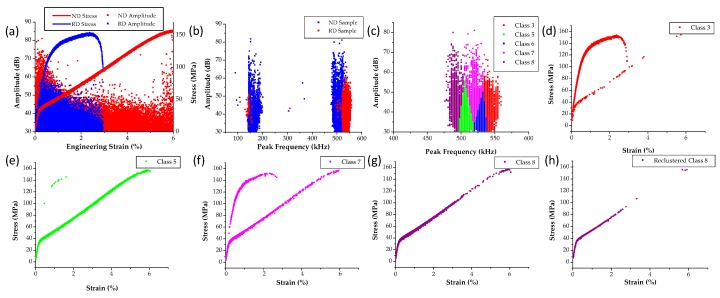
(**a**) Stress-strain curves and amplitude plot of ND and RD test samples at the MTS level; (**b**) frequency amplitude plot of both samples; (**c**) K-Means class distribution of data with peak frequency between 400 and 600 kHz; (**d**–**g**): class 3, 5, 7, 8 AE hits; (**h**) reclustered class 8 AE hits.

**Figure 13:**
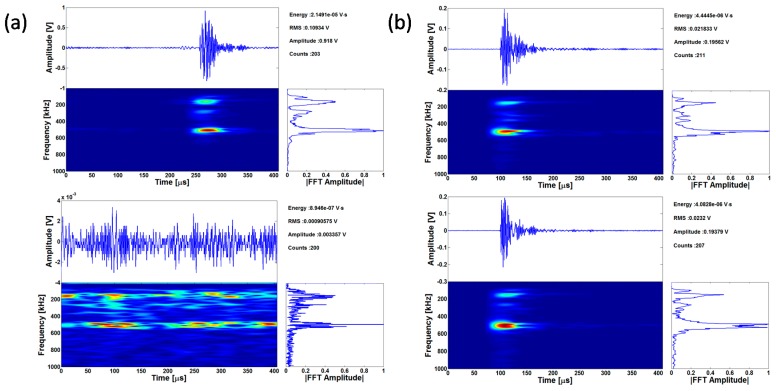
(**a**) Randomly selected signals from class 8 in Figure 12 showing both continuous and burst type emissions; (**b**) randomly selected signals from reclustered class 8 containing only burst emissions.

The procedure developed for the tensile tests inside the SEM was further applied to the MTS level tests using the specimens shown in Figure 2c. The authors have recently shown that this type of geometry due to the strain localization zone between the two notches twins profusely and therefore the AE datasets obtained could be used for comparisons with the tests inside the SEM [68]. The overall mechanical behavior for both ND and RD specimens combined with the AE activity is shown in Figure 12a, while Figure 12b shows the peak frequency of this AE activity. In this case amplitude, absolute energy, and peak frequency were chosen as the descriptors used in clustering. Again, eight classes were determined using the same procedure as described in the previous section and only these AE signals shown in Figure 12b with a peak frequency in the 400–600 kHz range. The clustering results are shown in Figure 12c–g. Using the same criteria described in the previous section, class 8 was identified as the twinning class and therefore it was reclustered to select only the burst type signals. Some representative waveforms of the original class 8 are shown in Figure 13, which contains both continuous and burst type waveforms. However, the reclustering procedure showed that only burst type signals can be found in the new class 8, similar to the procedure followed for the datasets obtained from testing inside the SEM.

### 3.4. Summary of AE Signals by Twinning in Magnesium

Based on the results presented in this section, a summary of AE activity characteristics associated with twinning is shown in Table 1. The results based on the clustering from the polycrystalline specimens (both inside the SEM and at the MTS level) are found to agree with the results obtained by single crystal testing (twin 1 and twin 2 referring to the highlighted AE hits in Figure 4a). Specifically, the AE signals identified as twin related were of burst nature with relative high (~500 kHz) peak frequency and frequency centroid values, as well as relatively high rise and decay angles. The high rise and decay angle of AE signals captured in this study agree with a previous report [13], as nucleation of twins produce burst type AE signals. The high frequency of twinning AE signals has also been previously reported [46,53,65]. Regarding their amplitude, it makes physical sense that the amplitude of AE signals in the single crystal tests is higher than the polycrystals, as the twins produced in the single crystal testing are in general larger and are activated easier since there is no obstacles (e.g., grain boundaries) or other effects (e.g., texture) which would affect the activation of this deformation mechanism. In contrast, twin activation in polycrystals is highly dependent on crystallographic orientation and grain size as previously shown by the authors [56,57]. 

The quantitative characteristics of AE signals from twinning are in agreement with previous studies [13,36,40,41,42,43,44,52,53,65,66]. To the best of the authors’ knowledge, no such characteristics of AE signals from twining have been reported directly. Furthermore, unlike previous machine learning attempts using adaptive sequential K-means method [53], this study uses a two-step K-means clustering to obtain those AE signals that are most probable to be associated with twinning. Such results further benefit from comparisons between Magnesium single crystal and polycrystal testing, which deviates from the AE analysis done previously [47].

## 4. Conclusions

The results in this article relate twinning activity across scales with Acoustic Emission information. To the best of the authors’ knowledge, this is the first time that testing of Magnesium single crystals is compared with both testing inside the scanning electron microscope and at the standard laboratory scale using the same type of Acoustic Emission monitoring system and paired, when possible, with full field deformation measurements. The mechanical test data presented in this article, directly show that twin activation at both single crystal and polycrystalline level is associated with distinct AE activity, which was cross-validated by the overall stress strain curve (by seeing load drops and stress plateau regions formed), as well as microscopy information. Such microscopy revealed twin boundary formation at locations where strain localizations appear in single crystal testing, as well as texture and in situ twin nucleation and growth in polycrystals tested inside the electron microscope. The availability of these datasets and the fact that such AE activity could be correlated directly with both mechanical test data and microscopy led to the application of a post processing method, which resulted to the definition of ranges for features of Acoustic Emission signals which appear to be related to twinning. This approach, provides evidence of the applicability and usefulness of nondestructive evaluation based on acoustics to assist characterization and monitoring of dominant deformation mechanisms in advanced materials. 

## Figures and Tables

**Table 1:** Comparison of AE features related to twinning activity in Magnesium.

AE Features of Twinning	Single Crystal Twin 1	Single Crystal Twin 2	SEM AZ31 Twin Clustering (388 hits)	MTS AZ31 Twin Clustering (410 hits)
Amplitude (dB)	74	75	69 ± 6	60 ± 8
Rise Angle	0.721	0.724	0.92 ± 0.41	0.51 ± 0.45
Decay Angle	0.838	0.913	0.89 ± 0.38	0.46 ± 0.42
Peak Frequency (kHz)	513	484	465 ± 37	507 ± 14
Frequency Centroid (kHz)	471	500	502 ± 70	520 ± 68
